# Long-Term Strength Development of Fly Ash-Based One-Part Alkali-Activated Binders

**DOI:** 10.3390/ma14154160

**Published:** 2021-07-27

**Authors:** Sani Haruna, Bashar S. Mohammed, Mohamed Mubarak A. Wahab, Mubarak Usman Kankia, Mugahed Amran, Abdurra’uf Mukhtar Gora

**Affiliations:** 1 Civil Engineering Department, Bayero University, Kano 700241, Nigeria; amgora.civ@buk.edu.ng; 2 Department of Civil and Environmental Engineering, Universiti Teknologi Petronas, Seri Iskandar 31750, Perak, Malaysia; mubarakwahab@utp.edu.my (M.M.A.W.); mubarak_18001828@utp.edu.my (M.U.K.); 3 Department of Civil Engineering, College of Engineering, Prince Sattam Bin Abdulaziz University, Alkharj 11942, Saudi Arabia; mugahed_amran@hotmail.com; 4 Department of Civil Engineering, Faculty of Engineering and IT, Amran University, Amran 9677, Yemen

**Keywords:** density, efflorescence formation, long-term strength, properties, one-part alkali-activated binders

## Abstract

This research aims to study the effect of the dosage of anhydrous sodium metasilicate activator on the long-term properties of fly ash-based one-part alkali-activated binders (OPAAB) cured at ambient conditions. Powdered sodium metasilicate activator was utilized in the range of 8–16% by weight of the fly ash in producing the OPAAB. The properties examined are hardened density, compressive strength, flexural strength, water absorption, efflorescence formation, and microstructural analysis. The experimental result revealed that the binders exhibited excellent long-term strength properties. The compressive strength of the OPAAP is well correlated with its hardened density. The pastes were found to exhibit good soundness characteristics over the long-term. The absorption of water decreases with an increase in the activator dosage from 8–12%, and beyond that, the water absorption relatively remains the same. Field emission scanning electron microscope (FESEM) micrograph revealed uniformly formed solid matrices with the micro-crack present were observed in the samples. The larger pore size promotes the crystallization of the resulting hydrate substances (N, C)-A-S-H gel. The initial dissolution of the OPAAP occurred within the first 30 min. At longer age of curing, mixtures with a higher dosage of powdered activator tend to absorb less water. Strength properties beyond 28 days are considered as the long-term strength.

## 1. Introduction

The excessive use of cement in concrete has created a lot of environmental concerns in terms of both the damage caused by the mining of the raw materials and carbon dioxide (CO_2_) emissions during cement production. It is evident that besides the depletion of natural resources in the production of Portland cement binders, a huge amount of carbon dioxide was discharged into the surrounding atmosphere. Concrete accounts for 5–8% of whole anthropogenic carbon dioxide emissions and most of the CO_2_ was emanated from cement production [1]. It is estimated that the world’s annual cement output of 2 billion tonnes releases around 1.65 billion tonnes of CO_2_, or approximately 7% of the overall greenhouse gas emissions into the atmosphere [2]. The rate at which the CO_2_ is discharged during the production of ordinary Portland cement (OPC) is one-in-one, that is for each 1 kg of OPC produced 1 kg of CO_2_ [1]. In lieu of this fact, the concrete industry is under pressure worldwide to find a substitute binding material that can mitigate the use of cement. It is, therefore, essential to replace Portland cement with low CO_2_ emission materials for the production of environmentally friendly concrete [3,4].

Portland cement generation increases the worldwide greenhouse gas emissions outflows through the calcination of clinker in hydrocarbon warmed heaters. Generally, a decrease in cement utilization has been accomplished by the utilization of industrial by-products, for example, fly ash (FA) and ground granulated blast furnace slag (GGBS) as partial or complete replacement materials to Portland cement in concrete. Nowadays, various regulatory standards of using alkali-activated materials have been proposed in a different part of the world, for small- and large-scale production [5,6]. For more than a century of intermittent use, the main motive behind the acceptance of alkali activation was the possible reduction of CO_2_ emissions. The essential factor that is likely to assess the possibility of alkali-activated binders being absorbed and used in any selected area is the abundance of adequate raw materials [7].

One-part alkali-activation is a new geopolymer development method that has been introduced to reduce the complexities of coping with alkaline solution enabled geopolymers by combining aluminosilicate precursors with powdered activators [8,9]. Unlike conventional geopolymer binders where solutions are used to activate the activation phase, the activator had been in dry powdered form in the one-part binder, and the reaction immediately started as water was poured into the binder like OPC binders [8,10,11]. This approach helps to prevent the use of corrosive and caustic solutions for mass production of geopolymer concrete. There are several efforts at creating one-part binders along with alkaline solutions at higher temperatures to synthesise aluminosilicate materials [12]. Compared to traditional geopolymers, one-part geopolymers have low environmental carbon emission as the polymerization phase makes up only a fraction of the framework [13]. They also found that the binding components of the one-part geopolymers are comparable to the two-part geopolymers. The manufacturing of one-part alkali-activated binders (OPAAB) consists of a dry mixture of a solid aluminosilicate precursor and a solid alkali activator, to which water is added, like the OPC preparation [14,15]. Over the past years, one-part geopolymers like OPC manufactured by simply adding water have attracted strong interest [16,17]. The production of OPAAB will enhance the commercial viability of the geopolymer and have the ability to considerably reduce CO_2_ emissions compared to OPC mixtures [2,18]. In OPAAB, dry blends consisting of aluminosilicate precursors and solid alkaline activators are required. The replacement of cement with cellulose nanocrystals is reported to have reduced the greenhouse gases emitted during cement production and enhanced the fracture behaviour of cementitious materials [19].

Anhydrous sodium metasilicate activator demonstrated an important activation effect in one-part alkali-activated materials. It exhibits excellent strength development at ambient curing. Ma et al. [20] have produced a one-part geopolymer with anhydrous sodium metasilicate and anhydrous sodium carbonate activators. They have reported a high early strength of 35 MPa at 1-day of ambient curing by activating slag with Na_2_SiO_3_-anhydrous. However, the addition of Na_2_CO_3_ reduces its mechanical strength. It was observed that the strength reduction is more significant at an early age than the longer age. Ahmad et al. [21] have also produce one-part alkali-activated mortars with anhydrous sodium metasilicate powder. They have found that the long-term strength (270 days) of the developed one-part alkali-activated mortars (OPAAM) was significantly higher than that of the two-part alkali-activated mortars. They have concluded that the addition of admixtures is insignificant in improving the mechanical properties of OPAAM.

Compared to cement-based mortars, alkali-activated materials (AAM) generally have much higher shrinkage. This is because water does not integrate directly into aluminosilicate gel production, unlike the OPC system, and a tiny amount of water stays as interstitial water. As such, a significant quantity of water is not chemically bound and thus likely to evaporate. AAM has shown practicable performance in building construction in terms of mechanical properties, but there are still problems with large-scale implementations of AAM. These problems include unknown shrinkage behaviour of various AAM [22]. During the drying process of cementitious materials, drying shrinkage occurs. It is the volume changes resulting from the expulsion of moisture from the surface of the gel pores [23]. Even though AAM is engaged in various chemical processes, it is more difficult to understand the shrinkage behaviour in these binders than composites from OPC. To investigate the effect of different parameters on the drying shrinkage of AAM, numerous studies have been carried out [24,25,26]. However, information about this binder systems shrinkage behaviours is dispersed and fragmented. Bakharev et al. [27] reported that heat treatment decreases the rate of drying shrinkage compared to ambient curing. However, using elevated temperature to reduce shrinkage is more pronounced in alkali-activated slag (AAS) binders than FA-based binders. Mastali et al. [25] have comprehensively documented that in the AAS/FA binders, the drying shrinkage often relies on the regimes of curing and the dosage of the activator. A ternary blend of slag, silica fume, and phyllite dust achieves a record high strength of 145 MPa of OPAAB [28].

Although several studies [13,20,21,29] have reported about the performance of one-part alkali-activated materials, most of these studies were performed using low calcium FA, slag, or FA/slag-based precursors. Most of the research works have concentrated on early age and 28 days of mechanical performance without considering the long-term longevity of the OPAAB. Therefore, this paper aims to investigate the long-term strength behaviour of FA-based OPAAB. Strength properties tested beyond 28 days are considered as the long-term strength in this paper. Dry powder geopolymer cement as a ready-to-use product that can be packed in bags and mixed with water like OPC was produced. This new product is likely to have high potential to become an alternative to OPC than the conventional geopolymers and will aid full utilization and commercialization of alkali-activated concrete. The developed binder can perfectly be used for repair and maintenance applications as well as underwater construction.

## 2. Materials and Methods

### 2.1. Materials

**Figure 1:**
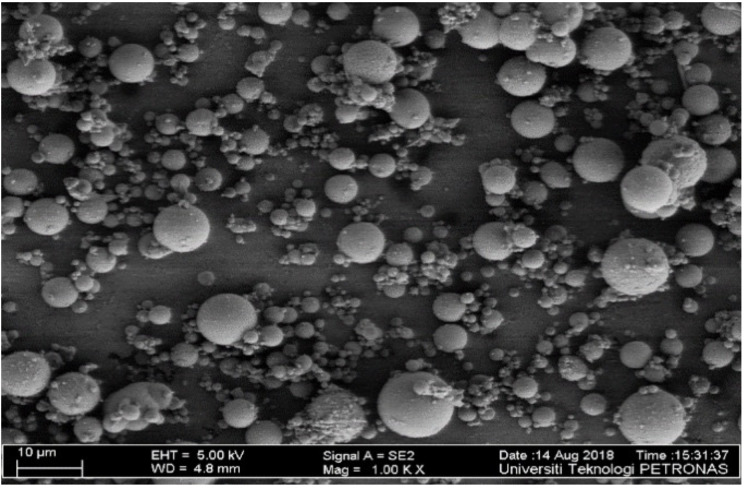
Field emission scanning electron microscope (FESEM) of fly ash (FA).

In this investigation, FA of high calcium content taken from a Manjung coal power plant, Perak, Malaysia was employed as the primary ingredient of aluminosilicate material. Anhydrous sodium metasilicate (ASMS) was obtained from Portray Sdn Bhd, Selangor, Malaysia. The activator was used as the solid activator, which is blended with the FA to form the binder. The oxides of the FA and ASMS were determined through X-ray Fluorescence (XRF) and presented in Table 1. The total percentage of Al_2_O_3_, Fe_2_O_3_, and SiO_2_ is 68.7% for FA; thus, the FA satisfies the requirement of class C FA as per ASTM 618-15 [30] and ASTM C305 [31]. It is well noted that CaO was more than 10% for high calcium FA, and its loss of ignition was 0.17%. The loss of ignition of 0.17% indicates that the FA has low combustible carbons. The FA has a specific gravity of 2.35 and Blaine fineness of 386 m^2^/kg. The morphology of the FA was determined using Field Emission Scanning Microscope (FESEM, SUPRA 66VP by Carl Zeiss, Jena, Germany) and presented in Figure 1. Based on the FESEM micrograph, all the FA particles appeared to be spherical and mostly amorphous, which enables them to blend freely in mixtures. The less water demand by the FA is attributed to its spherical shape.

### 2.2. Mixture Design and Specimen Preparation

The mixtures were designed based on the authors’ previous work, the powdered sodium metasilicate anhydrous was used as the component of the binder as shown in Table 2. 

The mixing process of the one-part alkali-activated binders has been conducted using a Hobart mortar mixer (Obtained from Sri Kirushna, Tamil Nadu, India) in compliance with the standard procedure of ASTM C305-14 [31]. The production of one-part geopolymer binders involves blending of the precursor materials with granular sodium metasilicate activator for about 3 min to obtain a uniform dry geopolymer binder. Progressively, clean tap water was injected into the dry mixture at a constant water-to-binder ratio of 0.25 and stirred for another 3 min until it was uniform and coherent. The mixing has been done in a laboratory at 25 °C. The mix design is in accordance with our previous work [14]. To obtain the compressive strength and water absorption, the mixtures were cast into 50 mm cubic moulds. Flexural beams of 160 mm × 40 mm × 40 mm were similarly cast for determining the flexural strength of the OPAAB. All the specimens were cured at the laboratory temperature of 25 °C.

### 2.3. Experimental Test Procedures and Setup

#### 2.3.1. Hardened Density Test

The hardened density of one-part alkali-activated binders as evaluated by weighing the cube specimens on the testing day prior to the compression tests in accordance with ASTM C642 [32].

#### 2.3.2. Compressive Strength Test

**Figure 2:**
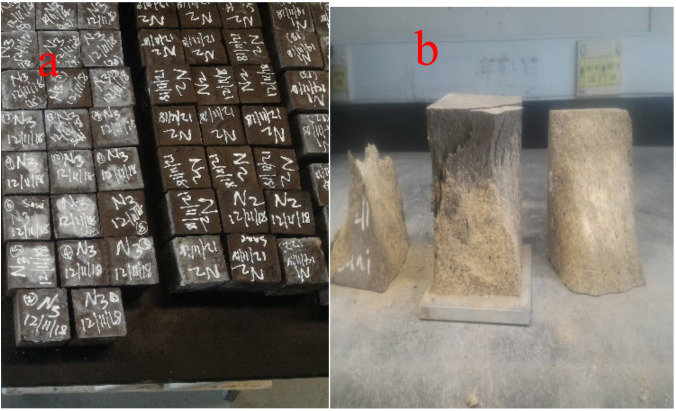
One-part alkali-activated binders (**a**) before test (**b**) after test.

On 50 mm cubes tested with a 3000 kN digital compression machine, the compressive strength test was performed. In compliance with BS 12390-3:2009 [33], the loading rate was set at 0.90 kN/s. The hardened cubes were measured at 28, 56, 90, 180, and 365 days for each combination, in which 3 samples were examined for each curing time, and the average result was reported as the compressive strength. The specimens before and after the test were shown in Figure 2a,b.

#### 2.3.3. Flexural Strength Test

Prismatic beams with dimensions of 160 mm × 40 mm × 40 mm were used to measure the flexural strength of the prismatic beams. The test was conducted in accordance with the requirements outlined in ASTM C293M-10 [34]. Six beams were produced for each mixture and tested at a period of 28 and 90-days. To determine the flexural strength of the samples subjected to the three-point bending test, Equation (1) [35] is used.

(1) $$\mathrm{Flexural}\;\mathrm{strength}=\frac{3\mathrm{FL}}{2{\mathrm{bh}}^{2}}$$

where F is a failure load, L is the active length of the beam, b and h is the beam width and height, respectively.

#### 2.3.4. Water Absorption Test

Hardened cubes specimens of size 50 mm × 50 mm × 50 mm have been used in conducting the water absorption test as per ASTM C642 [32] specifications. After the curing period had lapsed, the specimens were removed and dried in an oven at 105 °C for 24 h before being weighed. The specimens were returned to the oven at 105 °C for another 24 h before being cooled and weighed. The procedure was repeated until the difference between two consecutive weight measurements was less than 0.5% of the lowest value. The specimens were allowed to cool at laboratory temperature after being removed from the oven and weighed.

#### 2.3.5. Efflorescence Test

The efflorescence intensity of the aged specimens was observed by visual comparison. The efflorescence products are observable with the naked eye. There is currently no international standard or test method for determining the efflorescence degree of alkali-activated binders. The total CO_3_^2−^ mass-produced during efflorescence process is used in this study to characterize the efflorescence degree.

#### 2.3.6. Microstructural Investigations

To better understand the microstructural properties of the OPAAB, the specimens were analysed by a Field emission scanning electron microscope (FESEM, SUPRA 66VP by Carl Zeiss Jena, Germany). The FESEM test has been conducted in accordance with ASTM C1723-16 [36]. To assess heat flow modifications connected with material transformations, a differential scanning calorimetry test has been carried out as per ASTM E1269-11 [37].

## 3. Results and Discussion

### 3.1. Density of One-Part Alkali-Activated Binders

**Figure 3:**
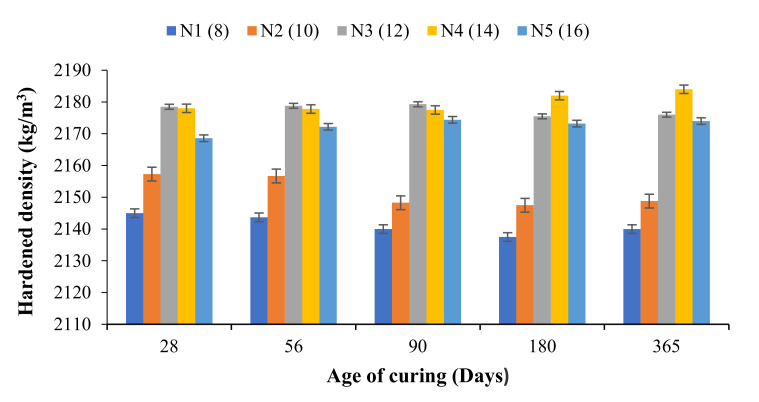
Hardened density of N1–N5 OPAAB.

From Figure 3, it can be observed that the hardened density of the developed OPAAB increased with increasing the amount of the solid activator. The hardened density was obtained in accordance with ASTM C 642 [32]. The hardened density increased linearly with the amount of sodium metasilicate activator in the mixes. The age of curing has a negligible effect on the densities of the developed OPAAB, which indicates good soundness of the developed OPAAB. The density of the OPAAB was in the range between 2150–2185 kg/m^3^. The values obtained were in reasonable agreement with that reported by [38]. The density of the N1 and N2 samples slightly decreases with age, which is associated with poor bonding between the FA particles. The poor bonding may also be attributed to lower alkali content within the mixture. The density of the pastes decreases slightly after 90 days of ambient healing. In general, it can be deduced that the hardened density of geopolymer paste depends on the specific gravity and fineness of the source materials used.

### 3.2. Compressive Strength of One-Part Alkali-Activated Binders

**Figure 4:**
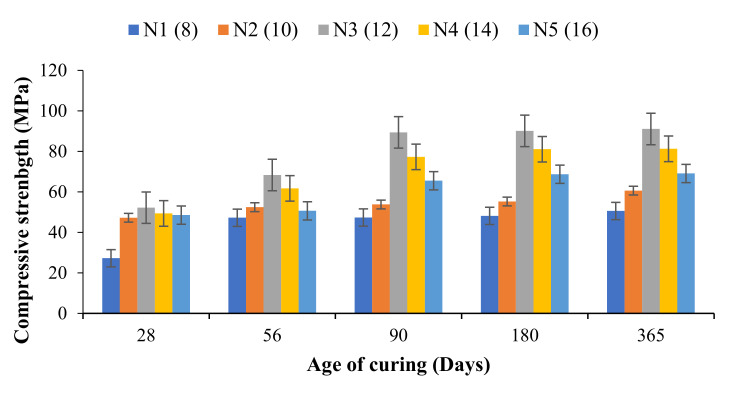
Compressive strength development of OPAAB.

Figure 4 represents the compressive strength growth of the OPAAB from 28 days up to 365 days. Compressive strength of 25–52 MPa was realised for N1 to N5 samples at 28 days of ambient curing. After 28 days, there is a continuous improvement in the strength performance for all the OPAAP mixtures. At 56 days, the compressive strength of OPAAB increases by 73.4% for N1, 11.1% for N2, 30.9% for N3, 25.1% for N4, and 4.3% for N5 specimens. At 90 days, the compressive strength was found to increase by 73.8, 13.9, 71.2, 56.6, and 34.5% for N1, N2, N3, N4, and N5. It is worth noting that the maximum strength was obtained at 90 days of ambient curing for all the mixtures. The strength enhancement at 180 and 365 days are very negligible compared to 28–90 days. From Figure 4, it was observed that N3 specimens have the highest compressive strength of almost 90 MPa at 90 days. The rate of strength growth at longer periods was evaluated by calculating the ratio of 365 days to 28 days’ strength, and the ratio was found to be higher for OPAAB than that of the conventional geopolymer paste. It is believed that the presence of excess sodium oxide in the mix slows down the strength growth slightly. The ratio of the 365 to 28 days of the control mix N1, N2, N3, N4, and N5 were 1.38, 1.81, 1.22, 1.76, 1.65, and 1.44. The emergence of C-A-S-H gel is suspected to contribute to the enhancement of the compressive strength [39,40]. The increase in strength beyond 28 days can be due to the formation of reaction of FA at a later age, thereby generating more calcium alumina silicate hydrate (C-A-S-H), thus enhancing the long-term strength. It was suspected that the C-A-S-H gel inside the OPAAB fills the voids and pores. This helps bridge the voids between the various hydrated phases and unreacted FA particles, resulting in improved compressive strength [41].

**Figure 5:**
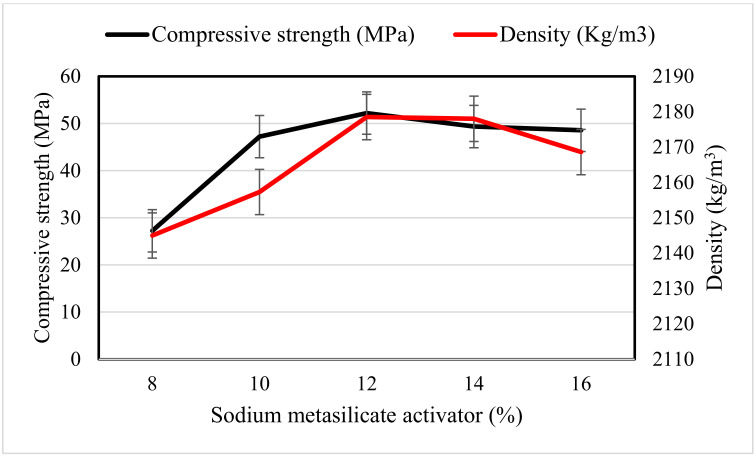
Compressive strength and density of OPAAB at 28 days.

The compressive strength and hardened densities of the OPAAB at 28 days are shown in Figure 5. It is worth mentioning that the hardened densities of the OPAAB complied with the compressive strength as it increases with the increase in activator content. The reduction in density at 16% of sodium metasilicate content is attributed to the presence of high efflorescence salt on the surface of the pastes, which created pores in the OPAAB matrix. The results were in good agreement with that of Nematollahi et al. [8].

### 3.3. Flexural Strength OPAAB 

**Figure 6:**
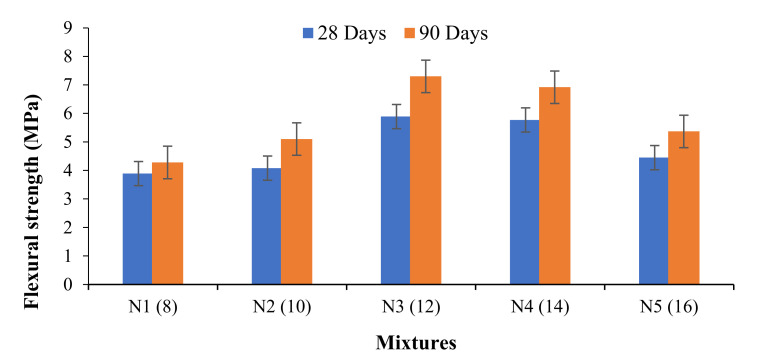
Flexural strength of OPAAB.

The flexural strength of the OPAAB specimens can be used as its tensile strength. Nevertheless, the flexural strength usually shows a higher strength result than the splitting tensile strength. Figure 6 shows the comparison of the flexural strengths between the control mixture of two-part geopolymer paste and the corresponding OPAAB having a different dosage of powdered activator. The flexural strength of the OPAAB shows a similar trend with that of its compressive strength, that is, it increases with age. It also increased with the increase in the activator dosage. The flexural strengths of the binders obtained at 28 days of ambient curing were 3.89, 4.08, 5.89, 5.77, and 4.45 MPa for N1, N2, N3, N4, and N5 mixtures. The flexural strength still improved after 90 days as compared to the 28-day strength. Compared to 28 days, the strength at 90 days improved significantly by 10, 25, 23.9, 19.9, and 20.7% for N1, N2, N3, N4, and N5 mixtures. The highest flexural strength of approximately 6 and 7.3 MPa was realized for N3 mixtures at 28 and 90 days of ambient curing. The result is well correlated with the work of Luukkonen et al. [42]. However, they have achieved maximum flexural strength with the aid of plastic sealing curing technique. Prolonging the curing age beyond 90 days had no significant effect on the strength development at ambient curing. The flexural strength is well correlated with its corresponding compressive strength result.

### 3.4. Extent of Efflorescence Formation of OPAAB

**Figure 7:**
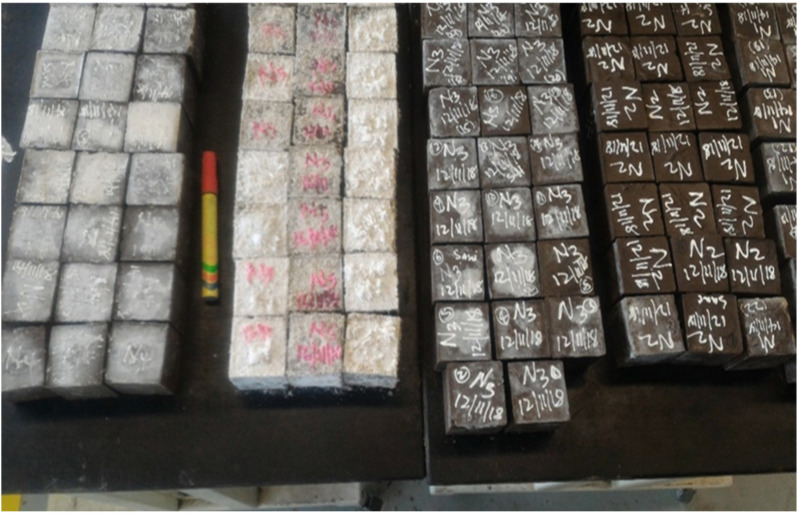
Extent of efflorescence formation of OPAAP.

It is well established that the appearance of efflorescence in OPAAB is primarily due to the interaction of atmospheric CO_2_ with excess soluble alkali on the surface of the resulting geopolymer binders when the concrete products are subjected to moist air or when they come into contact with water [43]. In this study, the OPAAB samples were manufactured at laboratory temperature without covering them with anything or sealing them. Figure 7 shows visible efflorescence formations observed in some of the OPAAB samples. As presented in Figure 7, the leaching of sodium is directly proportional to the dosage of the activator. As observed in the hardened OPAAB, mixtures with higher molar ratios of Na/Al demonstrate a higher degree of alkali leaching, signifying a higher tendency to efflorescence. This matches the established pattern in the chemistry of porous solutions as a function of alkali content, where higher Na/Al ratios give more alkaline porous formulations. The efflorescence formation mechanism in geopolymer binders can be represented in the terms given in Equations (2) and (3).

(2) $$CO_{2(g)}+2OH^{-}{}_{\left(\mathrm{aq}\right)}\;\overset{\;}{\to }\;CO_{3}{}^{2-}{}_{\left(\mathrm{aq}\right)}+H_{2}O$$

(3) $$2Na^{+}{}_{\left(\mathrm{aq}\right)}+CO_{3}{}^{2-}{}_{\left(\mathrm{aq}\right)}+H_{2}O\;\overset{\;}{\to }\;{\mathrm{Na}}_{2}{\mathrm{CO}}_{3}\cdot \;H_{2}O_{\left(s\right)}$$

As demonstrated in Figure 7, the presence of efflorescence in the N1 and N2 series specimens was not witnessed, although efflorescence was visible in the N4 and N5 series specimens. This observation can be attributed to the presence of excess sodium ions in the system, which did not completely react with the source materials. Therefore, the sodium ions react with the carbonate and formed sodium carbonate hydrate, which deposited on the surface of the specimens as presented in Equation (3) [44]. For N3 specimens, that is, samples made with 12% sodium metasilicate activator, the efflorescence formation on the surface of the specimens was not significant. This discovery can be justified by the presence of higher quantities of FA in the mix, resulting in the complete use of the granular activator during the geopolymerization process.

### 3.5. Water Absorption

**Figure 8:**
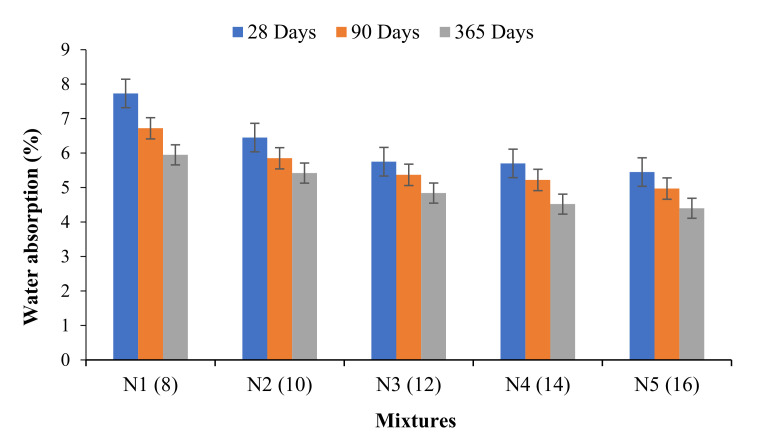
Water absorption of OPAAB.

The test was performed at 28, 90, and 365 days. At 28 days of ambient curing, the rate of water absorption of one-part alkali-activated binders recorded in this study was evaluated in the range of 5.45–7.73%. For the same mixtures, the decrease in water absorption was observed in the range of 4–15% and 16.2–29.9% at 90 and 365 days. The reduction of water absorption from 28–90 days for the designed mixtures N1, N2, N3, N4, and N5 was found to be 15%, 10.3%, 7%, 9.2%, and 9.7%, respectively. The reduction in water absorption continued to decrease further from 28–365 days by 29.9%, 19%, 16.2%, 22.2% and 23.9%. It can be inferred from Figure 8 that with increased activator dosage, water absorption decreases and vice versa. The mixture of 8% ASMS explicitly indicates a very high absorption of water by capillary movements. It is important to note that the absorption of water was proportional to the geopolymeric ingredients and the dosage of the activator, the greater the dosage of the activator, the more resistance to water penetration, and consequently the less harm to the substance caused to the surface. The absorption of water decreases with an increase in the activator dosage from 8–12%, and beyond that, the water absorption relatively remains the same. The reduction in water absorption at a higher dosage of the activator is because of the pore enhancement of the pastes. However, at longer age of curing, mixes with higher activators tend to absorb less water. This is related to the refinement of the pore due to the total disintegration of the powdered anhydrous-Na_2_SiO_3_ that filled the micro crack that formed in the initial reaction process. It was observed that the water absorption of all samples was below the 10% threshold suggested by Neville [45]. Moreover, it was observed that at all ages the minimum water absorption was achieved in the range of 12–16% of the activator dosage.

### 3.6. Field Emission Scanning Electron Microscopy Analysis

**Figure 9:**
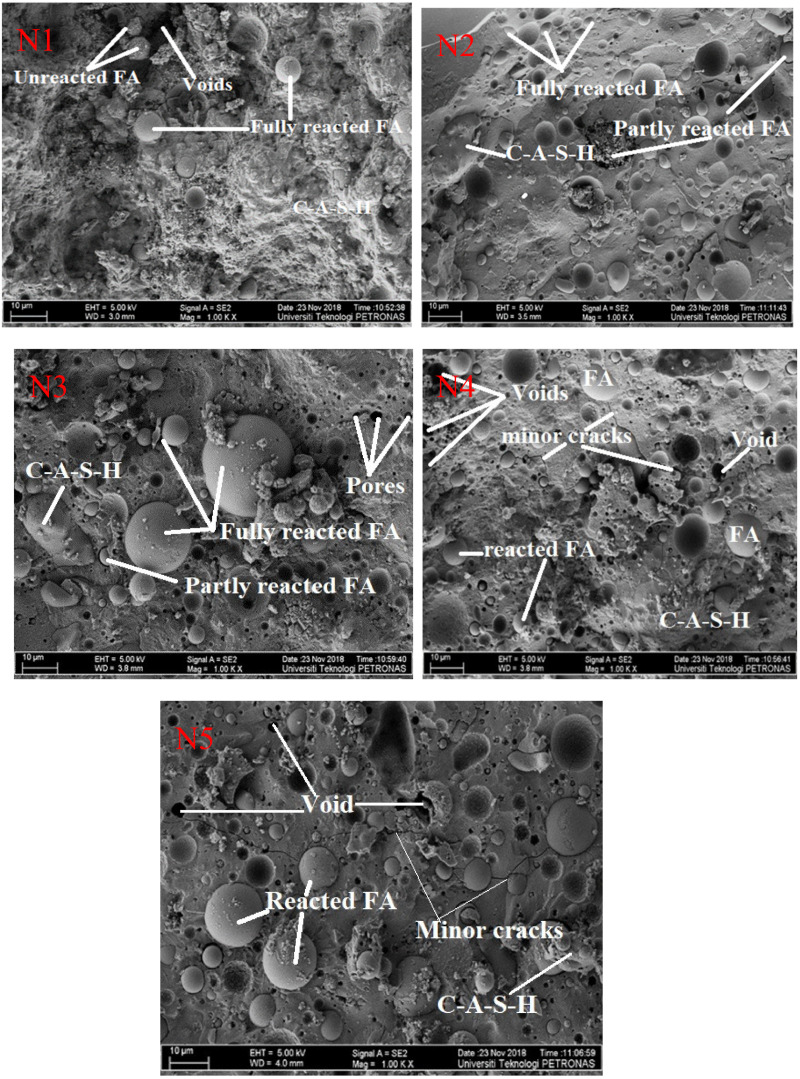
Field Emission Scanning Electron Microscopy Micrograph at different dosage of solid activator.

Figure 9 shows the FESEM micrographs of the OPAAB at a different dosage of anhydrous sodium metasilicate activator. The N1 sample shows a less dense microstructure that is consistent with its lower strength than the N2, N3, N4, and N5 specimens. N3 has a microstructure with the highest packing density and few unreacted FA particles that contribute to enhanced strength. N4 and N5 have many unreacted FA particles with fewer micro-cracks and voids. The voids could be related to the heat released during the dissolution of the anhydrous sodium metasilicate activator. Longer micro-cracks are more pronounced in N5 samples compared to the other specimens, which can be related to the higher dosage of the activator. Similar behaviour was reported by Ma et al. [20]. Uniformly formed solid matrices with the micro-crack present were observed in the samples; the larger pore size promotes the crystallization of the resulting hydrate substances (N, C)-A-S-H gel. As the binder used consisted primarily of Class C FA and this amorphous precursor is rich in calcium, calcium aluminium silicate hydrate (C-A-S-H) gel is therefore assumed primarily to form in the matrix. At a higher dosage of the sodium metasilicate, the concentration of ionic species increased, limiting the mobility of the ions, and delaying the formation of coagulated structures. This can interrupt the formation of the one-part alkali-activated paste [46]. This can be due to excess Na_2_O in the system, which generates heat during the geopolymerization process at the higher activator, resulting in the creation of small cracks on the specimens’ surface. Such cracks are correlated with heat developing during the reaction phase. Moreover, there are fewer unreacted FA particles in the matrix, which is suspected to the enhancement of the strength at higher relatively high dosage of the anhydrous sodium metasilicate activator.

### 3.7. DSC Analysis

**Figure 10:**
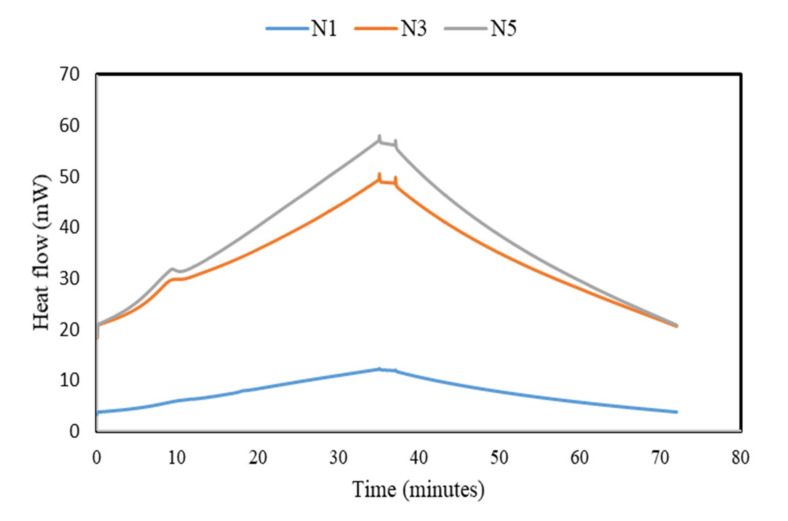
Heat evolution of OPAAB.

Figure 10 demonstrates the DSC analysis thermogram of one-part alkali-activated binder. The initial dissolution of the OPAAB occurred within the first 30 min. It is worth mentioning that the heat flow for FA-based OPAAB is dependent on the dosage of anhydrous sodium metasilicate activator. The rate of heat release for N1 mixtures was less than 10 mW, which is very low compared with N3 and N5. The highest heat flow of about 55 and 50 mW occurred at 35 min for N3 and N5 mixtures. As the dosage of the activator increases, the intensity of the initial peak is considerably increased, implying a more intensive dissolution and reaction process. The rapid increase at the initial stage is associated with the initial wetting and dissolution of FA in the alkaline medium. The high heat of reaction is associated with the lower alkali modulus of the activator used [47]. The solid activator dissolves immediately after adding water, producing a large amount of heat and releases a high concentration of OH^−^ and (SiO_4_)^4^ [20]. Higher alkalinity also enhances the solubility of silica and alumina in the mixtures that could aid the production of reaction products. It is interesting to note that after about 40 min, the heat release decreases continuously and stabilizes after 70 min. This confirmed that the reaction mechanism of a high calcium-based one-part geopolymer is rapid, and the high energy released during the reaction process contributed to the quick setting time of the OPAAB. The activator dosage has a significant impact on the duration of the induction period and contributed significantly to the total heat release.

## 4. Conclusions

The performance of one-part alkali-activated materials has been reported by previous studies, but most of these studies were performed using low calcium FA, slag, or FA/slag-based precursors. Most of the research works have concentrated on early age and 28 days of mechanical performance without considering the long-term longevity of the OPAAB. Therefore, this study systematically focuses on the effect of the dosage of anhydrous sodium metasilicate activator on the long-term strength properties and drying shrinkage of FA-based one-part alkali-activated binders cured at ambient conditions. The heat of the reaction was also analysed with the aid of differential scanning calorimetry. Based on the results of the experimental results of this study, the following conclusions can be outlined below,

- The hardened densities of the developed one-part alkali-activated pastes increase with the increase in activator content. The binders were found to exhibit good soundness characteristics over the long-term.
- After 28 days of ambient curing, the strength behaviour of the one-part alkali-activated binders improved drastically. The developed OPAAB achieved its peak strength at 90 days of ambient curing. The compressive strength of the binder significantly enhanced by 74% from 28 days to 1 year. The 90 days compressive strength of OPAAP activated with 12% Na_2_SiO_3_-anhydrous can reach up to 90 MPa. Strength increment at 180 and 365 days is negligible.
- The water absorption of the OPAAB decreases with the age of curing. Over a longer term, the water absorption of the binders reduced by 16–30% for all the samples.
- The efflorescence of the OPAAB rises with an increase in the amount of anhydrous-Na_2_SiO_3_ activator and age of curing. At a lower dose of the powdered activator, the efflorescence can be avoided. The efflorescence at a high dose of activator interferes with the reaction process, thereby slightly affecting its strength growth.
- Increasing the dosage of the anhydrous sodium metasilicate activator increases the initial heat release rate. No significant exothermic reactions were observed at a lower dose of anhydrous sodium metasilicate activator.
- Dry powder geopolymer cement as a ready-to-use product that can be packed in bags and mixed with water like OPC was developed. This new product is likely to have a high potential to become an alternative to OPC than the conventional geopolymers.

## Figures and Tables

**Table 1:** Chemical composition of the binder materials (percentage by weight).

Oxide Compositions	Fly Ash (FA)	Anhydrous-Na_2_SiO_3_
SiO_2_	37.3	46
Al_2_O_3_	14.90	-
Fe_2_O_3_	16.5	-
CaO	17.9	-
MgO	2.08	-
SO_3_	0.7	-
K_2_O	2.8	-
Na_2_O	0.26	51
TiO_2_	1.07	-
MnO	0.13	-
H_2_O	-	3
LOI	0.17	-

LOI: Loss of ignition.

**Table 2:** Mix proportion of one-part alkali-activated binders.

Mixtures	FA (%)	Na_2_SiO_3_ Anhydrous (%)	w/b Ratio
N1	92	8	0.25
N2	90	10	0.25
N3	88	12	0.25
N4	86	14	0.25
N5	84	16	0.25

w/b—Water-to-binder.

## Data Availability

All the data is available within the manuscript.
